# How Valid Are Cortisol and Galvanic Skin Responses in Measuring Student Stress During Training? Comment on the Psychological Effects of Simulation Training

**DOI:** 10.2196/45340

**Published:** 2023-08-18

**Authors:** Urvi Sonawane, Pragna Kasetti

**Affiliations:** 1 Imperial College London London United Kingdom

**Keywords:** augmented reality, AR, salivary cortisol, galvanic skin conductance, medical simulation, medical education

We read with great interest the article “Comparing the Psychological Effects of Manikin-Based and Augmented Reality–Based Simulation Training: Within-Subjects Crossover Study” by Toohey et al [1]. We commend the authors for considering medical students’ psychological well-being and the risk of excessive stress in the advent of augmented reality (AR) exploration. However, we wish to discuss certain aspects of the research.

First, the time point of salivary cortisol measurements, at 15 minutes post simulation, may not be sufficient, as cortisol levels peak approximately 30 minutes after a stressful event [2]. Hence, the traumatic scenario ending of pediatric death may not be captured in this last cortisol measurement, underestimating the stressful impact of the scenario. In addition, interperson variability is exacerbated by factors including smoking, coffee, and alcohol consumption [2]. Hence, measurement or controlling of these factors prior to simulation may aid in the accuracy of results. Moreover, as nearly one-third of individuals do not mount a cortisol response [2], markers such as α-amylase, as done by Stecz et al [3], may be considered in the future.

Comparable stress responses between AR and manikin-based simulations are promising for the future of AR in medical teaching. However, we are concerned about the validity of the galvanic skin response (GSR) measurement, especially as it was the only finding that differed between both simulations. Participants may have had excess palmar sweat or products interfering with the GSR measurement (eg, hand lotions); this was not addressed in the protocol through prior handwashing [4]. Postsimulation GSR measurements may also be worthwhile to observe because the stress during personal postsimulation reflection has not been considered.

Furthermore, student demographic characteristics, including socioeconomic background and ethnicity are not detailed. Members of racial and ethnic minority groups and the working class experience greater chronic stress and cumulative stress exposure during their lives [2]. As such, these characteristics are suggested to influence physiological and psychological stress responses [2]. Hence, these potential confounders should be detailed and adjusted so that the study results are considered in the context of wider student populations. Determining the representativeness of the student sample would also be aided by detailing the proportion of participants with pre-existing psychological traits (ie, depression and posttraumatic stress disorder).

The implications of this study for future research are promising. Stecz et al [3] measured heart rate variability and blood pressure, which could be useful, as greater cardiovascular responses to stress increase long-term cardiovascular risk. Furthermore, it could also be valuable to have further descriptions of the students’ opinions regarding which simulation fulfilled their learning outcomes better. Additionally, knowing student perspectives on whether a certain scenario suited one type of simulation more than the other can explore the nuances of simulations; it may be that one modality is not best for all scenarios.

In conclusion, the authors have conducted a valuable and needed study in the face of the ever-growing field of AR. However, we highlight recommendations regarding outcome measurements, demographics, and avenues for future exploration.

## Abbreviations

AR: augmented reality
GSR: galvanic skin response

## References

[1] Toohey S, Wray A, Hunter J, Waldrop I, Saadat S, Boysen-Osborn M, Sudario G, Smart J, Wiechmann W, Pressman SD (2022). Comparing the psychological effects of manikin-based and augmented reality-based simulation training: within-subjects crossover study. JMIR Med Educ.

[2] Crosswell AD, Lockwood KG (2020). Best practices for stress measurement: how to measure psychological stress in health research. Health Psychol Open.

[3] Stecz P, Makara-Studzińska M, Białka S, Misiołek H (2021). Stress responses in high-fidelity simulation among anesthesiology students. Sci Rep.

[4] Villanueva I, Valladares M, Goodridge W (2016). Use of galvanic skin responses, salivary biomarkers, and self-reports to assess undergraduate student performance during a laboratory exam activity. J Vis Exp.

